# Human protein interaction networks across tissues and diseases

**DOI:** 10.3389/fgene.2015.00257

**Published:** 2015-08-19

**Authors:** Esti Yeger-Lotem, Roded Sharan

**Affiliations:** ^1^Department of Clinical Biochemistry and Pharmacology, Ben-Gurion University of the NegevBeer-Sheva, Israel; ^2^Blavatnik School of Computer Science, Tel Aviv UniversityTel Aviv, Israel

**Keywords:** protein interaction network, tissue-specific network, disease-specific network, network perturbation, gene expression

## Abstract

Protein interaction networks are an important framework for studying protein function, cellular processes, and genotype-to-phenotype relationships. While our view of the human interaction network is constantly expanding, less is known about networks that form in biologically important contexts such as within distinct tissues or in disease conditions. Here we review efforts to characterize these networks and to harness them to gain insights into the molecular mechanisms underlying human disease.

## Introduction

Protein molecules constitute the main building blocks of cells and mediate most cellular processes. In human, they are encoded by over 22,000 different genes, which give rise to many more proteins through alternative splicing mechanisms. These numerous proteins do not work in isolation: instead, they interact with each other and with other types of molecules to form complex cellular machines and to pass signals within cells and across tissues. In recognition of the fundamental role of these molecular interactions, much effort has been invested in the last two decades in their mapping. From small-scale experiments that measure interactions between a few proteins, mapping has changed to large-scale screens using high-throughput techniques such as yeast two-hybrid and co-immunoprecipitation (e.g., Rual et al., 2005; Stelzl et al., 2005; Ewing et al., 2007; Rolland et al., 2014). Owing to these mapping efforts, our current view of the physical interactions between human proteins encompasses over 200,000 interactions among over 20,000 proteins, and is continuously expanding. The resulting network of all known protein-protein interactions (PPIs), known as the human interactome, has become a key framework for studying protein function, cellular processes, and genotype-to-phenotype relationships, as reviewed elsewhere (Barabási et al., 2011; Vidal et al., 2011). However, this broad network is also limited. PPIs have rarely been measured in the context of distinct cell types, tissues, or in disease conditions, making it difficult to model and understand context-related phenotypes.

While knowledge of human context-specific PPIs is limited, we are witnessing a rapid accumulation of context-specific molecular expression profiles. The human body consists of tens of tissues, sub-tissues, and cell types that differ from one another in morphology and function. In a seminal study published more than a decade ago, Su et al. (2004) opened a window into their molecular characteristics by profiling the transcriptomes of 79 human tissues via DNA microarrays. Other studies profiled the transcriptomes of human tissues by techniques such as massively parallel signature sequencing (Jongeneel et al., 2005), expressed sequence tags (EST) (Hillier et al., 1996), and next generation RNA sequencing (e.g., Illumina's BodyMap 2.0). Most recent is the RNA sequencing of multiple human tissues from a number of individuals by the Genotype Tissue Expression project (Mele et al., 2015). The proteomes of human tissues have also been profiled by immunohistochemistry (Pontén et al., 2009; Uhlén et al., 2015) and mass-spectrometry techniques (Kim et al., 2014; Wilhelm et al., 2014). In addition to efforts to profile normal tissues, profiling techniques have also been employed to characterize different diseases. One of the more prominent initiatives is The Cancer Genome Atlas (TCGA) (Weinstein et al., 2013), which is actively mapping genomic, transcriptomic, proteomic, and epigenomic changes in cancerous tissues compared to normal tissues. These measurements shed light on the parts of the interactome that are active in these diverse contexts, although direct experimentation is required to reveal the actual PPI changes, in particular the formation of novel interactions (Ideker and Krogan, 2012). Below we discuss efforts to harness these context-specific molecular expression profiles to elucidate network properties of human tissues and to identify interaction-based disease mechanisms.

## Features of tissue and cell-type specific networks

**Figure 1:**
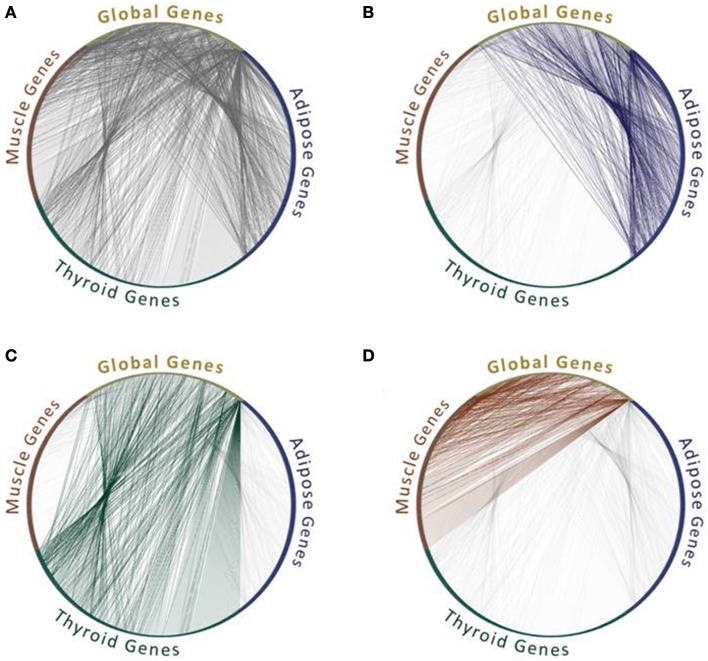
**Feasible protein interactions change between tissues**. All protein interactions **(A)** and feasible protein interactions that connect “global genes,” which are expressed in all three tissues, with tissue-specific genes that are expressed in one tissue out of adipose **(B)**, or thyroid **(C)**, or muscle **(D)**. Data of the genes expressed per tissue were extracted from GTEx Portal (Mele et al., 2015) and limited to genes with 50 counts and above. Data of protein interactions were extracted using MyProteinNet (Basha et al., 2015) from BioGrid (Chatr-Aryamontri et al., 2015), DIP (Xenarios et al., 2002), IntAct (Kerrien et al., 2012), and MINT (Licata et al., 2012) databases. Only global genes that have tissue-specific interactions in each of the three tissues are shown.

Given the lack of context-specific PPIs that were measured in different tissues and cell types, many studies revert to identifying PPIs that are feasible in these contexts. Their underlying assumption is that a PPI is feasible within a specific context if the corresponding proteins are expressed in that context. Of course not all feasible interactions actually take place, as they depend on many other factors such as localization and conformation of the two proteins, yet co-expression is necessary. Additionally, co-expression has often been based on RNA levels, as protein expression levels were rarely available. This approach had been used previously in model organisms to analyze their network dynamics in response to stimuli (Luscombe et al., 2004) or during cell cycle (de Lichtenberg et al., 2005), and has been used extensively for analyzing tissue interactomes (e.g., Lopes et al., 2011; Barshir et al., 2013; Song et al., 2014). Some differences in the sets of PPIs that are feasible within tissues and involve tissue-specific (TS) proteins and globally expressed (GE) “housekeeping” proteins are exemplified in Figure 1.

One of the first questions that had been asked was whether genes and PPIs that appear to be TS or GE have distinct topological features relative to the generic human interactome or to each other. Dezso et al. (2008) complied transcriptome profiles of 31 tissues, and found that the set of GE genes was larger than previously assumed. They showed that the topology of the GE PPI network was characterized by higher connectivity and shorter paths between proteins relative to the generic interactome. Lin et al. (2009) analyzed the number of interactions (degree), closeness, and betweenness centralities of GE and TS proteins within the generic PPI network. They found that GE genes were more central and may form a core, while clusters of TS genes attach to the core at more peripheral positions in the network. Using the data of Su et al. (2004), Bossi and Lehner (2009) found extensive direct interactions between GE and TS proteins, and suggested a model for the evolution of TS functions through the modification of core cellular processes. Souiai et al. (2011) used EST data across 45 tissues to test whether tissue-specificity is encoded in the interactome. They also found that GE genes were located at the topological center of the interactome. Denoting interactions occurring at a subset of tissues as TS interactions (TSI), they found that TSI involved in regulatory and developmental functions were also central, whereas TSI involved in organ physiological functions were peripheral. Kiran et al. (Kiran and Nagarajaram, 2013) analyzed features of highly connected proteins, namely hubs, in tissue interactomes. They showed that, among other features, TS hubs were associated with a lower degree of interactome centrality as compared with GE hubs. Waldman et al. (2010) analyzed translation efficiency, and showed that genes that were translated more efficiently in a specific tissue encode proteins that tend to have more interactions in that tissue, relative to other proteins in the same tissue.

The application of RNA-sequencing to human tissues revealed that many more transcripts were expressed per tissue than previously acknowledged (Ramsköld et al., 2009). Emig and Albrecht (2011) were among the first to harness RNA-sequencing data to the analysis of tissue interactomes. They showed that, in contrast to previous studies based on microarray profiles, TSI were less common, and were mainly involved in transmembrane transport and receptor activation. They also suggested that a considerable part of tissue-specificity is likely to be achieved by alternative splicing and interactions involving protein isoforms (further discussed in Buljan et al., 2012). In accordance with this suggestion, Ellis et al. (2012) demonstrated experimentally that neural-regulated exons can remodel PPIs by stimulating and repressing different partner interactions. Another study showed that proteins enriched with splice variants tend to occupy central positions in tissue interactomes (Sinha and Nagarajaram, 2014). Recently, it was claimed that splicing play mostly a complementary role in driving cellular specificity, except for the brain, which exhibits a more divergent splicing program (Mele et al., 2015).

Another technological breakthrough that is taking place in recent years is the profiling of proteomes at large scale. Since the correlation between transcript and protein levels is partial (Schwanhausser et al., 2011), proteome profiling opens a more direct way to identify feasible PPIs. Liu et al. (2014) used proteomic data (Kim et al., 2014) to analyze tissue interactomes. They showed that, relative to the generic interactome, tissue interactomes are smaller, sparser, and that hubs may have more important roles. Barshir et al. (2013) combined transcript and protein measurements to create 16 extensive tissue interactomes. Their comparative analysis (Barshir et al., 2014) revealed that each tissue interactome is dominated by a core sub-network that is common to all tissues, with only a small fraction being TS. Most tissue hubs were GE and retained their large PPI degree across tissues, and were enriched in regulatory functions. Lastly, they found in each tissue a significant correlation between transcript expression level and number of PPIs involving the encoded protein.

An important application of tissue interactomes is to shed light on disease mechanisms. Lage et al. (2008) systematically mapped over 1000 heritable diseases to the tissues in which they manifest clinically by using text-mining. They showed that proteins and complexes that were linked to diseases tend to be over-expressed in the tissue where defects cause pathology, with the exception of proteins and complexes associated with cancers. Magger et al. (2012) showed that the usage of tissue interactomes, created from a generic interactome by removing or penalizing interactions involving non-expressed proteins, considerably improved the prioritization of disease genes. Li et al. (2014) assessed tissue interactomes weighted by DNA methylation data, and showed that they enhance prediction of disease genes. Barshir et al. (2014) focused on genes causing hereditary diseases and found that they tend to have PPIs that occur exclusively in the tissue where defects cause pathology. They demonstrated that these tissue-exclusive PPIs can highlight disease mechanisms, and, owing to their small number, suggested that they constitute an efficient filter for interrogating disease etiologies.

## Perturbed networks in disease

Protein networks are perturbed in disease due to sequence mutations and expression changes. Zhong et al. (2009) were the first to systematically probe the effect of sequence (disease-causing) mutations on PPIs. They focused on known mutations causing Mendelian disorders and categorized them according to whether they have a truncation effect (“truncating,” including nonsense mutations, out-of-frame indels, or defective splicing) or not (“in-frame,” including missense mutations and in-frame indels). They showed that truncating mutations seem to lead to node-removal effects in the PPI network, while in-frame mutations are associated with edge-specific perturbations.

In a later study, Wang et al. (2012) examined the effect of disease-causing mutations using a structurally resolved PPI network, consisting of interactions and their atomic-resolution interfaces. They found that in-frame mutations tend to occur on the interaction interfaces of causal proteins and no similar enrichment was detected in non-interacting domains. This suggests that PPI perturbations play an important role in disease. Additionally, they found that the disease specificity for different mutations on the same gene can be explained by their location within the interface, further underscoring the importance of PPIs for the study of disease mechanisms.

On the technological side, Wei and Yu (Wei et al., 2014) developed an experimental pipeline to examine the consequences of different mutations on protein stability and interactions. They used the pipeline to show that disease causing mutations on interactions interfaces are more likely to perturb the corresponding interactions than mutations away from interfaces. Lambert et al. (2013) developed an experimental pipeline to score modulated interactions. The pipeline couples affinity purification to data-independent mass-spectrometric acquisition. The authors used it to identify interaction changes following disease-associated mutations and drug exposure.

Recently, Rolland et al. (2014) compared the impact of mutations associated with human disorders to that of common variants with no reported phenotypic consequences on PPIs. They focused on 32 genes with 115 disease and common variants, testing up to four disease and four common variants per disease gene for their impact on the ability of the corresponding proteins to interact with known interaction partners. They found that disease variants were 10-fold more likely to perturb interactions than common variants; more than 55% of the 107 interactions tested were perturbed by at least one disease-associated variant. In a follow-up study, Sahni et al. (2015) investigated the consequences of 2890 disease-causing missense mutations in 1140 genes. Out of 197 mutations covering 89 proteins with at least two PPI partners (in the HI-II-14 map of Rolland et al., 2014), 26% were found to cause a complete loss of interactions, 31% resulted in specific loss of some interactions, and 43% did not change the interaction partners. Disease mutations were shown to perturb interactions that are functionally relevant in the particular tissue affected by the specific disease. Sahni et al. further conclude that gain of interactions is a rare event in human disease, finding very little evidence for it.

## The road ahead

Network biology in the past decade was focused on general networks per species, representing the interaction potential of every two proteins. It is becoming clear that these networks, while providing important insights, do not materialize in all conditions. Rather, different sub-networks are formed in different contexts depending on protein expression, structure, and more. In the future, when interactome measurements become as standard and inexpensive as genome sequencing, one can envision the construction of patient-specific networks that could dramatically improve our understanding of human disease and its treatment. With the accumulation of more individual-specific network data, statistical techniques that are currently limited to sequence data, such as association studies, could be generalized to the network world, ever refining our views of cells and organisms.

### Conflict of interest statement

The authors declare that the research was conducted in the absence of any commercial or financial relationships that could be construed as a potential conflict of interest.
